# Leishmanicidal compounds of *Nectria pseudotrichia*, an endophytic fungus isolated from the plant *Caesalpinia echinata* (Brazilwood)

**DOI:** 10.1590/0074-02760170217

**Published:** 2018-02

**Authors:** Betania Barros Cota, Luiza Guimarães Tunes, Daniela Nabak Bueno Maia, Jonas Pereira Ramos, Djalma Menezes de Oliveira, Markus Kohlhoff, Tânia Maria de Almeida Alves, Elaine Maria Souza-Fagundes, Fernanda Fraga Campos, Carlos Leomar Zani

**Affiliations:** 1 Fundação Oswaldo Cruz-Fiocruz, Instituto René Rachou, Laboratório de Química de Produtos Naturais Bioativos, Belo Horizonte, MG, Brasil; 2 Fundação Oswaldo Cruz-Fiocruz, Instituto René Rachou, Laboratório de Genômica Funcional e Proteômica de Leishmania spp. e Trypanosoma cruzi, Belo Horizonte, MG, Brasil; 3 Universidade Federal de Minas Gerais, Departamento de Fisiologia e Biofísica, Belo Horizonte, MG, Brasil; 4 Universidade Estadual do Sudoeste da Bahia, Departamento de Química, Jequié, BA, Brasil; 5 Universidade Federal dos Vales do Jequitinhonha e Mucuri, Departamento de Ciências Biológicas e da Saúde, Diamantina, MG, Brasil

**Keywords:** fungal metabolites, natural products, Leishmania braziliensis, antileishmanial, neglected disease

## Abstract

**BACKGROUND:**

In a screen of extracts from plants and fungi to detect antileishmanial activity, we found that the ethyl acetate extract of the fungus *Nectria pseudotrichia*, isolated from the tree *Caesalpinia echinata* (Brazilwood), is a promising source of bioactive compounds.

**OBJECTIVES:**

The aims of this study were to isolate and determine the chemical structures of the compounds responsible for the antileishmanial activity of the organic extract from *N. pseudotrichia*.

**METHODS:**

Compounds were isolated by chromatographic fractionation using semi-preparative high-performance liquid chromatography, and their chemical structures were determined by analytical and spectral data and by comparison with published data. The antileishmanial activity of the isolated compounds was evaluated in intracellular amastigote forms of *Leishmania (Viannia) braziliensis* expressing firefly luciferase as reporter gene, and cytotoxicity was determined in Vero and THP-1 mammalian cell lines by MTT assay.

**FINDINGS:**

Fractionation of the extract yielded seven compounds: 10-acetyl trichoderonic acid A (1), 6′-acetoxy-piliformic acid (2), 5′,6′-dehydropiliformic acid (3), piliformic acid (4), hydroheptelidic acid (5), xylaric acid D (6), and cytochalasin D (7). Compounds 1, 2 and 3 are reported here for the first time. Compounds 1, 2, and 5 were more active, with IC_50_ values of 21.4, 28.3, and 24.8 µM, respectively, and showed low toxicity to Vero and THP-1 cells.

**MAIN CONCLUSIONS:**

*N. pseudotrichia* produces secondary metabolites that are more toxic to intracellular amastigote forms of *L. (V.) braziliensis* than to mammalian cells.

Leishmaniasis is a group of human diseases caused by protozoan species of the genus *Leishmania*, which are prevalent in tropical and subtropical areas of the world (WHO 2010). Brazil is among the ten countries affected by 90% of the cases worldwide of both cutaneous and visceral leishmaniasis (WHO 2010).

*Leishmania (Viannia) braziliensis* is the main etiological agent of American tegumentary leishmaniasis (ATL) and has highest incidence in Brazil. This group of infectious diseases has different clinical forms that are associated with the molecular diversity of the parasite and host immune response (Pereira et al. 2017). The control of ATL has been based on chemotherapy with pentavalent antimonials for more than 70 years. Meglumine antimoniate (Glucantime®) is a first-line drug, but use of this therapeutic is limited by its high cost and toxicity. Although there is little evidence of treatment failure for Glucantime® in Brazil (Nassif et al. 2016), the use of pentavalent antimonials are contraindicated in pregnancy and in patients with heart or renal disease, and they require daily parenteral administration. Thus, there is a need for the discovery of new leads or scaffolds that can be used to develop less toxic drugs and alternative oral treatments (Prates et al. 2017).

As part of our program to investigate endophytic fungi as sources of natural products with biological activities, particularly against neglected infectious diseases, we found that the ethyl acetate (EtOAc) extract from the culture of *Nectria pseudotrichia* (Nectriaceae), an ascomycete isolated from the tree *Caesalpinia echinata* (Fabaceae), was active against amastigote-like forms of *Leishmania (Leishmania) amazonensis* (Campos et al. 2015), showing an IC_50_ value of 4.6 µg/mL. Although the genus *Nectria* is known to produce a wide range of secondary metabolites (Parisot et al. 1983, Irvine et al. 2008, Wang et al. 2012), there are no reports on the isolation of natural products from *N. pseudotrichia*. These facts prompted us to pursue the isolation and identification of the compound(s) responsible for the observed antileishmanial activity, and contribute to knowledge of the chemistry of this fungus. In addition, we report herein three novel compounds (1-3) for the first time.

## MATERIALS AND METHODS

*Fungal material* - The stems of *C. echinata* Lam. (Fabaceae) were collected by JO Rego, IR Andrade, ACO Nogueira, and MGC Fernandes at the Zoo-Botanical Foundation, Belo Horizonte (FZB-BH), state of Minas Gerais, Brazil. A voucher specimen was deposited at Fundação Zoo-Botânica Herbarium under the code BHZB-6458. A sample of the isolated fungus was deposited at the Microorganisms and Cells Collection of the Federal University of Minas Gerais, Brazil. It was identified as *N. pseudotrichia* (Schwein.) Berk & MA Curtis (Nectriaceae) based on the internal transcribed spacer (ITS1-5.8S-ITS2) rDNA analysis using universal fungal primers, and the sequences were deposited in GenBank with the accession number KF611677 (Campos et al. 2015).

*Fermentation and extraction* - Two-mm-diameter plugs of endophytic fungus culture were inoculated into the centres of 90 Petri dishes (90 mm diameter) containing 20 mL malt extract agar medium (malt extract 1% w/v, glucose 1% w/v, peptone 0.1% w/v and agar 1% w/v in 1 L purified water) and incubated at 25 ± 2°C for 14 days. The cultures were extracted three times by maceration with EtOAc for 48 h at room temperature. After passing through a filter paper, the solvents were evaporated at 45°C under reduced pressure in a rotary-evaporator. Residual solvent in the extracts was eliminated in a vacuum centrifuge at 40°C to yield 2.83 g of crude extract.

*Chromatographic fractionation* - Analytical high-performance liquid chromatography (HPLC) was performed on a Shim-pack® C18 column (5 µM, 4.6 mm i.d. x 250 mm), using a chromatograph equipped with LC10AD pumps, a mixing valve, and an SPD M-10A VP Diode Array Detector (Shimadzu, Kyoto, Japan). Semi preparative purifications were performed on a Shim-pack® C18 (5 µM, 20 mm i.d. x 250 mm) column using a chromatograph with two LC6AD pumps and an SPD-10A-UV detector (Shimadzu). Thin-layer chromatography (TLC) analyses were conducted on pre-coated silica gel G-60/F254 plates (0.25 mm, Merck, Darmstadt, Germany). The spots were visualized after heating the plate sprayed with a mixture of equal parts of ethanol solutions of vanillin (1% w/v) and sulphuric acid (10% v/v). Medium pressure column chromatography (MPLC) was performed over C18 silica gel (20-40 µM particle size) using a step gradient from 10 to 100% MeOH in water and collecting 136 fractions of 50 mL each. Fractions were pooled into 33 groups according to the similarity of their behaviour on TLC. Thus, group 12 (105 mg) was separated on a RP-C18 column using a 60 min linear gradient of 10 to 60% of MeOH in water, stepping to 100% MeOH and proceeding with this solvent for 10 min, yielding compounds 1 (18.9 mg), 5 (4.1 mg), and 6 (4.9 mg). Group 13 (300 mg) was also purified by a semi-preparative RP-C18 column, using a 60 min linear gradient from 50 to 100% MeOH in water, and held at 100% for 30 min, providing compounds 2 (32.1 mg) and 3 (39.0 mg). In the same way, group 14 (149 mg), after a 60 min linear gradient from 10 to 100 % MeOH in water, yielded compound 7 (20.2 mg), while group 15 (130 mg) provided compound 4 (9.4 mg).

*Analytical methods* - Optical rotations were recorded using an Anton Paar MCP 300 polarimeter (Anton Paar, Graz, Austria). UV spectra were obtained in a SPD M-10A VP Diode Array Detector (Shimadzu). 1D and 2D NMR experiments were acquired with a Bruker Avance 400 MHz spectrometer (Bruker Daltonics, Bremen, Germany) using TMS as an internal standard. High-resolution mass spectral data were obtained on a maXis ETD ESI-QTOF (Bruker Daltonics) coupled to a Nexera UH-PLC-system (Shimadzu) using a reverse phase column (Shim-Pack XR-ODSIII, 2.2 µM particle diameter, 2.1 x 200 mm, i.d) at 40°C under a flow rate of 200 µL/min. The mobile phases were 0.1% v/v formic acid in (A) water and (B) ACN. A mixture of 5% B was pumped for 0.5 min followed by a linear gradient from 5 to 100% B over 12.5 min and a hold at 100% B for 1 min. The following conditions were used: end plate offset -500 V; capillary voltage 4500 V; nebuliser pressure 2.0 bar; dry gas (nitrogen) flow rate 8.0 L/min; dry gas temperature 200°C. Data-dependent precursor fragmentation was performed at collision energies of 40 eV. Ion cooler settings were optimised within a 40-1000 *m/z* range using a solution of 10 mm sodium formate in 50% aq. 2-propanol as calibrant. Mass calibration was achieved by initial in-source infusion of 20 µL calibrant solution and post-acquisition recalibration of the raw data.

*Analytical and spectral data of isolated compounds* - Compound 1: colourless oil. ${\left[\alpha \right]}_{\text{D}}^{25}$ + 51.0 (*c* 1.7, MeOH). UV: transparent above l 220 nm. HRMS: [M+H]^+^, *m/z* 341.1593 (C_17_H_24_O_7_, 0.6 ppm) NMR data are shown in Table I.

**TABLE I:** NMR Spectroscopic Data (400 MHz in CD_3_OD) for compound 1

C position	δ_C_ (mult.)	δ_H_ (mult., *J* Hz)	HMBC*
1	50.98, CH	2.64 (d, 10.1)	2, 5, 6, 10, 14
2	177.85, C		
3	57.61, CH_2_	a: 4.28 (d, 12.3) b: 4.16 (d, 12.3)	4, 5, 17 4, 5, 17
4	135.30, C		
5	145.29, CH	6.68 (d, 10.5)	3, 4, 7, 17
6	40.58, CH	2.86 (ddd, 10.5, 10.5, 10.1)	1, 2, 4, 5, 7
7	45.87, CH	1.44 (tt like, 10.5, 3.0)	
8	21.29, CH_2_	1.80 (m)	6, 10
9	28.75, CH_2_	eq: 2.44 (dt br., 14.0, 4.4, 4.4) ax: 2.07 (m)	1, 10
10	86.24, C		
11	29.49, CH	1.75 (m)	
12	16.31, CH_3_	0.78 (d, 6.8)	7, 11, 13
13	21.96, CH_3_	0.98 (d, 6.8)	7, 11, 12
14	73.30, CH_2_	4.66 (d, 11.0) 4.55 (d, 11.0)	1, 2, 10 2, 9, 10
15	172.00, C		
16	21.91, CH_3_	2.02, (s)	10, 14, 15
17	170.34, C		

* the numbers correspond to carbon atoms as shown in Fig. 2.

Compound 2: pale yellow gum. ${\left[\alpha \right]}_{\text{D}}^{25}$ + 30.0 (*c* 1.6, MeOH). UV: transparent above l 220 nm. HRMS: [M+H]^+^, *m/z* 273.1334 (C_13_H_20_O_6_, 0.4 ppm). NMR data are shown in Table II.

**TABLE II:** NMR Spectroscopic Data (400 MHz in CD_3_OD) for compound 2

	Compound 2		
C position	δ_C_ (mult.)	δ_H_ (mult., *J* Hz)	HMBC*
1	170.23, C		
2	134.65, C		
3	39.01, CH	3.63 (q, 7.1)	2, 4, 1′, 7′
4	177.99, C		
1′	144.89, CH	6.82 (t, 7.6)	1, 2, 3, 1′, 2′
2′	29.48, CH_2_	a: 2.23 (dddd, 14.0, 7.6, 7.0, 7.0) b: 2.28 (dddd, 14.0, 7.6, 7.0,7.0)	2, 1′, 3′
3′	29.44, CH_2_	1.53 (quin like, 7.1)	1′, 2′, 4′
4′	26.85, CH_2_	1.43 (m)	3′
5′	29.64, CH_2_	1.66 (quin like, 7.2, 6.6)	3′, 4′, 6′
6′	65.68, CH_2_	4.06 (dd, 6.6)	4′, 5′, 1″
7′	16.50, CH_3_	1.30 (d, 7.1)	2, 3, 4
1″	173.23, C		
2″	20.97, CH_3_	2.02 (s)	1″

* the numbers correspond to carbon atoms as shown in Fig. 2.

Compound 3: white powder. ${\left[\alpha \right]}_{\text{D}}^{25}$ + 24.0 (*c* 2.0, MeOH). UV: transparent above l 220 nm. HRMS: [M+H]^+^, *m/z* 213.1121 (C_11_H_16_O_4_, 0.1 ppm). NMR data are shown in Table III.

**TABLE III:** NMR Spectroscopic Data (400 MHz in CD_3_OD) for compound 3

	Compound 3		
C position	δ_C_ (mult.)	δ_H_ (mult., *J* Hz)	HMBC*
1	170.06, C		
2	134.61, C		
3	38.90, CH	3.62 (q, 7.1)	1, 2, 4, 1′, 7′
4	177.87, C		
1′	144.84, CH	6.84 (t, 7.6)	1, 2, 3, 1′, 2′
2′	28.97, CH_2_	a: 2.22 (dddd, 14.0, 7.6,7.0, 7.0) b: 2.27 (dddd,14.0, 7.6, 7.0, 7.0)	2, 1′, 4′
3′	29.18, CH_2_	1.59 (quin like, 7.6, 7.3)	1′, 4′, 5′
4′	34.51, CH_2_	2.11 (qt, 7.2, 1.5)	3′, 5′, 6′
5′	139.52, CH	5.83 (ddt, *J* = 17.1, 10.2, 7.0, 7.0)	4′
6′	115.65, CH_2_	a: 5.03 (ddt, *J* = 17.1, 2.0, 1.7, 1.7) b: 4.97 (ddt, *J* = 10.2, 2.0, 1.7, 1.7)	4′, 5′ 4′, 5′
7′	16.44, CH_3_	1.30 (d, 7.1)	2, 3, 4

* the numbers correspond to carbon atoms as shown in Fig. 2.

*Assays with L. (V.) braziliensis* - *L. (V.) braziliensis* (strain MHOM/BR/1994/H3227) promastigotes were maintained in minimum essential culture medium (*α*-MEM) (Gibco/Brl, Grand Island, NY, USA) supplemented with 10% (v/v) heat inactivated foetal bovine serum (FBS, Cultilab, Campinas, SP, Brazil), 100 mg/mL kanamycin, 50 mg/mL ampicillin, 2 mm L-glutamine, 5 mg/mL hemin, and 5 mm biopterin (Sigma-Aldrich, St. Louis, USA), at pH 7.0 and incubated at 25°C.

For this assay, we used *L. (V.) braziliensis* LUC expressing firefly luciferase as a reporter gene. The transfection was performed as previously described (Roy et al. 2000). Human monocyte-derived macrophage cell line, THP-1, was maintained in RPMI 1640 medium supplemented with 10% FBS and differentiated in the presence of 20 ng/mL phorbol myristate for 72 h at 37°C. Cells were infected with *L. (V.) braziliensis* LUC promastigotes at a parasite/macrophage ratio of 10:1 for 3 h in a white walled 96-well tissue culture plate. Non-internalised parasites were removed by five washes with HEPES/NaCl buffer (20 mm HEPES, 0.15 M NaCl, 10 mm glucose, pH 7.2). The infected cells were then treated with 1.5-200.0 µg/mL of sample-tests and 0.006-0.8 µM of amphotericin B (positive control). After 72 h, RPMI was aspirated and luciferase activity was assessed by adding 20 µL reconstituted One-Glo™ Luciferase Assay system solution as enzyme substrate, following the manufacturer's instructions (Promega, Madison, WI, USA). Luciferase activity was measured by luminescence detection in a SpectraMax M5 luminometer (Molecular Devices, Sunnyvale, CA, USA) using 1 s integration/well. Non-infected THP-1 macrophages were used as signal background while non-treated infected THP-1 macrophages were used as control for growth comparison. Antileishmanial activity was expressed as the concentration required to inhibit 50% of parasite growth, as calculated by sigmoidal regression analyses using GraphPad Prism (Version 6.0c, GraphPad Software Inc., La Jolla, CA, USA).

To assess toxicity of the compounds on the host cells, THP-1 macrophages were seeded in 96-well plates at a density of 2 × 10^5^ cells per well, differentiated in the presence of 20 ng/mL phorbol myristate for 72 h at 37°C, and treated with 1.5-200.0 µg/mL of the compounds or 1.5-200.0 μM of Amphotericin B. After 72 h treatment with compounds, cell death was estimated using MTT, which is metabolised by viable cells resulting in a purple product that can be quantified using a spectrophotometer (absorbance at 570 nm). The results were calculated from the absorbance measurements using the percentage of cell death in relation to the untreated cell controls.

*Cytotoxicity assay using Vero cells* - African green monkey kidney cells (Vero) were used as model of normal cells and were provided by Erna Kroon (Federal University of Minas Gerais, Brazil). The cells were maintained in DMEM medium (Sigma-Aldrich), containing 5% FBS (Gibco/Brl), enriched with 1% antibiotic solution (100 IU/mL penicillin and 100 µg/mL streptomycin, Gibco/Brl). Vero cells were inoculated in 96 well plates at 1 × 10^4^ cells/well and the plates incubated for 24 h at 37°C. The samples and amphotericin B (positive control) were dissolved in DMSO (Sigma-Aldrich). The half maximal inhibitory concentration (IC_50_) was determined over a range of concentrations (9 nonserial dilutions: 200.0 to 0.78 µM). All cell cultures were incubated in a 5% CO_2_/95% humidified air atmosphere at 37°C for 72 h. Wells with 0.5% v/v DMSO were used as a negative control. All medium and reagents used in the assays were endotoxin free. Cell viability was estimated by measuring the mitochondrial reduction of MTT (Monks et al. 1991). The samples were tested in triplicate in two independent experiments. The results were expressed as percentage of viability in relation to the negative control (DMSO, 0.5% v/v), calculated as follows: percentage of cell viability (%) = [(treated mean OD / negative control) x 100] and obtained from two independent experiments performed in triplicate. The IC_50_ values were calculated by non-linear regression using GraphPad Prism® Version 5.01 software. The selective index (SI) was calculated by dividing IC_50_ Vero cells/IC_50_ *L. (V.) braziliensis*.

## RESULTS

**Fig. 1:**
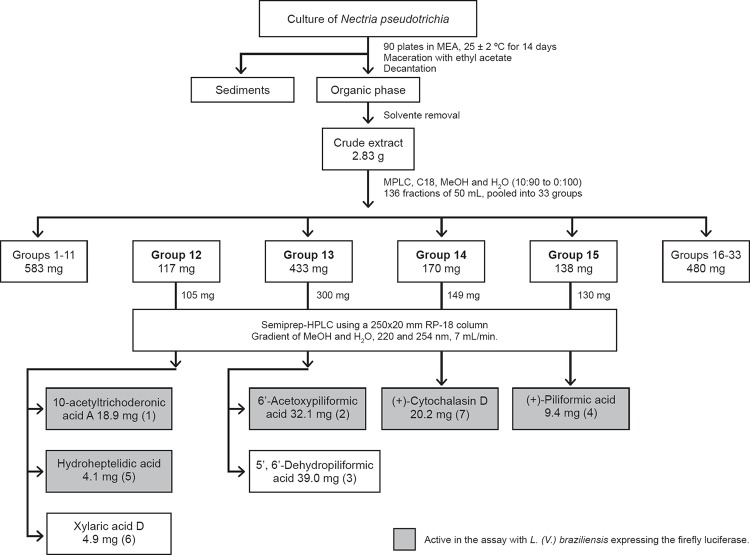
fractionation steps indicating the procedures, yields, and the active compounds in the assay with amastigote forms of *Leishmania* (*Viannia*) *braziliensis*.

Fig. 1 summarises the chromatographic fractionation steps used to isolate compounds 1-7 from crude EtOAc extract of *N. pseudotrichia*. Among the seven isolated compounds, three (1-3) are reported here for the first time.

**Fig. 2:**
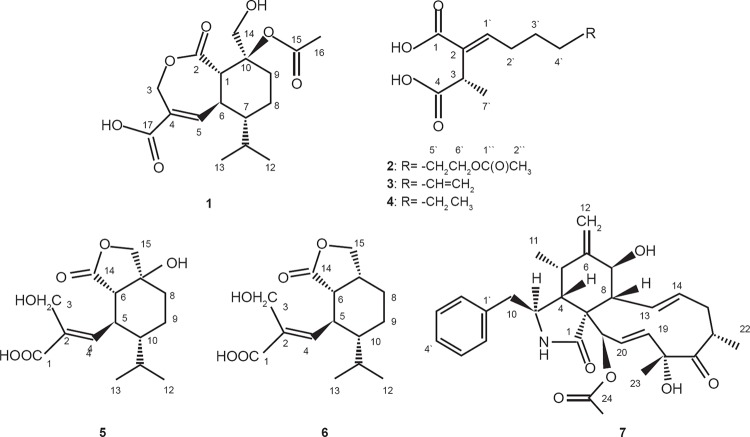
structures of compounds 1-7 isolated from the endophytic fungus *Nectria pseudotrichia*.

Compound 1 (Fig. 2) was isolated as a colourless oil. Its HRMS spectrum showed a [M+H]^+^ peak with *m/z* 341.1593, compatible with the molecular formula C_17_H_24_O_7_. The ^1^H- and ^13^C-NMR data (Table I) of one were very similar to those of trichoderonic acid A and (+)-heptelidic acid (Yamaguchi et al. 2010). However, one displayed signals δ_H_ 2.02 and δ_C_ 172.00 that were attributed to an acetyl group, which on the basis of HMBC correlations between H-10 and C-15, was allocated to C-10. The large coupling constants between H-1 and H-6, and H-6, and H-7 suggest an anti-orientation between them, indicating that the relative configurations at these positions are the same as in trichoderonic acid A (Yamaguchi et al. 2010). The NOESY experiment showed correlations between H-6 and H-11, H-12, H-13, and H-14. It also showed correlations between H-1 and H-7, corroborating the relative configuration shown in Fig. 2 (Yan et al. 2011). Furthermore, as both compound 1 and trichoderonic acid A have dextrorotatory activities, we propose that their absolute configuration is the same. Thus, one was identified as (+)-(5a*S*,6*R*,9*S*,9a*S*)-9-(acetyloxy)-9-(hydroxymethyl)-1-oxo-6-(propan-2-yl)-1,3,5a,6,7,8,9,9a-octahydro-2-benzoxepine-4-carboxylic acid, namely 10-acetyltrichoderonic acid.

Compound 2 (Fig. 2) was isolated as a pale yellow gum. Its molecular formula was determined by ESIHRMS to be C_13_H_19_O_6_. The compound showed ^1^H- and ^13^C-NMR data (Table II) similar to those of (+)-piliformic acid (4) (Anderson et al. 1985), with the major differences being the signals due the presence of an acetoxy moiety (δ_H_ 2.02s, δ_C_ 20.97 and δ_C_ 173.23) which, on the basis of HMBC cross peaks between H-6′ (δ_H_ 4.06) with the carbonyl of the acetoxy group (δ_C_ 173.23), led us to position this group at end of the aliphatic chain. The (*E*)-configuration was supported by NOESY correlations between H-3 (δ_H_ 3.63) and H-2′ (δ_H_ 2.23 and δ_H_ 2.28). Compound 2 showed dextrorotatory activity (${\left[\alpha \right]}_{\text{D}}^{25}$ + 30.0) as did the synthetic compound 2(*S*)-methyl-pent(*E*)-3-(methoxycarbonyl)-3-enoic acid (${\left[\alpha \right]}_{\text{D}}^{25}$ + 109) synthesised by Takashi et al. (1973) and for which the absolute configuration was established unambiguously. Thus, the absolute configuration of two was established (Fig. 2) and the compound identified as (+)-6′-acetoxy-piliformic acid.

Compound 3 (Fig. 2) was isolated as a white powder, and its molecular formula was determined to be C_11_H_16_O_4_ by HRMS. As for compound 2, its ^1^H and ^13^C NMR spectra (Table III) were similar to those of (+)-piliformic acid (4) (Table IV), however it showed one extra degree of unsaturation. Indeed, ^1^H and ^13^C NMR showed signals confirming the presence of an extra double bond (Table III) in the structure. This double bond was positioned at the end of the aliphatic chain on the basis of the observed COSY correlations between protons H-5′ with H-6′ and H-4′, and the HMBC correlations between proton H-4′ with carbons C-5′ and C-6′. The NOESY correlation between H-3 and H-2′, and the dextrorotatory activity of three indicates that it has the same absolute configuration as (+)-piliformic acid (4) (Table IV). Based on this evidence, the chemical structure of three is proposed to be (+)-(2*E*,3*S*)-2-(hept-6-en-1-ylidene)-3-methylsuccinic acid, and named 5′,6′-dehydro piliformic acid.

**TABLE IV:** NMR Spectroscopic Data (400 MHz in CD_3_OD) for compound 4

	Compound 4		
C position	δ_C_ (mult.)	δ_H_ (mult., *J* Hz)	HMBC*
1	170.08, C		
2	134.26, C		
3	38.81, CH	3.62 (q, 7.1)	1, 2, 4, 1′, 7′
4	177.88, C		
1′	145.16, CH	6.83 (t, 7.7)	1, 2, 3, 1′, 2′
2′	29.48, CH_2_	a: 2.21 (dddd, 14.7, 7.7, 7.2, 7.2) b: 2.26 (dddd, 14.7, 7.7, 7.2, 7.2)	2, 1′
3′	29.48, CH_2_	1.49 (quin like, 7.2)	1′, 4′, 5′
4′	32.71, CH_2_	1.36 (m)	
5′	23.57, CH_2_	1.33 (m)	
6′	14.36, CH_3_	0.92 (t, 7.0)	4′, 5′
7′	16.37, CH_3_	1.30 (d, 7.1)	2, 3, 4

* the numbers correspond to carbon atoms as shown in Fig. 2.

Analytical and spectral data of compounds 4-7 (Fig. 2) were identical to those published in the literature for (+)-piliformic acid (4) (Anderson et al. 1985), hydroheptelidic acid (5) (Calhoun et al. 1992, Yan et al. 2011), xylaric acid D (6) (Yan et al. 2011), and (+)-cytochalasin D (7) (Cafeu et al. 2005).

All compounds were tested against amastigotes forms of *L. (V.) braziliensis* (Table V; Supplementary data, Fig. 1) and compounds 1, 2, and 5 were more active, with IC_50_ values of 21.4, 28.3, and 24.8 µM, respectively. Compounds 4 and 7 showed IC_50_ values of 78.5 and 72.6 µM, respectively. None of these compounds were cytotoxic to THP-1 and Vero cells in the concentration range tested (Table V; Supplementary data, Figs 2-3). The IC_50_ values for amphotericin B on Vero and THP-1 cells were 18.2 and 12.0 µM, respectively. Considering that the IC_50_ values for all compounds were greater than 200 µM towards Vero and THP-1 cells (Table V), the selectivity indexes ranged from 2.5 (compound 4) to 9.3 (compound 1).

**TABLE V:** Activity of the isolated compounds 1-7 on intracellular amastigote forms of *Leishmania* (*Viannia*) *braziliensis*, Vero and THP-1 cell lines

Sample	*L.* (*V.*) *braziliensis* IC_50_ (µM)	THP-1 IC_50_ (µM)	Vero IC_50_ (µM)
1	21.4	> 200	> 200
2	28.3	> 200	> 200
3	> 200	> 200	> 200
4	78.5	> 200	> 200
5	24.8	> 200	> 200
6	> 200	> 200	> 200
7	72.6	> 200	> 200
Amphotericin B	0.12	12.0	18.2

## DISCUSSION

In our previous study, the crude extract from the culture of *N. pseudotrichia* disclosed antileishmanial activity without apparent toxicity to human peripheral blood mononuclear cells (Campos et al. 2015). These results prompted us to pursue the isolation and identification of the active metabolites in the extract (Fig. 1–2). Thus, three compounds were considered active when tested on intracellular amastigote forms of *L. (V.) braziliensis*: 6′-acetoxy-piliformic acid (2), piliformic acid (4), and hydroheptelidic acid (5).

The IC_50_ values of isolated compounds 1, 2, and 5 in our study were ~ 10 times lower than the IC_50_ values determined for meglumine antimoniate (178.2-330.2 µM) and paromomycin (233.6-344.4 µM), but ~ 5 times higher than the IC_50_ values determined for miltefosine (0.8-5.4 µM) and azithromycin (5.7 µM), and ~ 1000 times higher than the IC_50_ values obtained for amphotericin B (0.02-0.06 µM) in the assay with amastigote forms of *L. (V.) braziliensis* strain MHOM/BR/75/M2903 using different time periods of drug exposure (three, five, and seven days) (de Morais-Teixeira et al. 2014). Moreover, the IC_50_ value for amphotericin B (0.12 µM), used as positive control in our assays, was also ~ 200 times lower than the IC_50_ determined for compounds 2, 4, and 5.

**Fig. 3:**
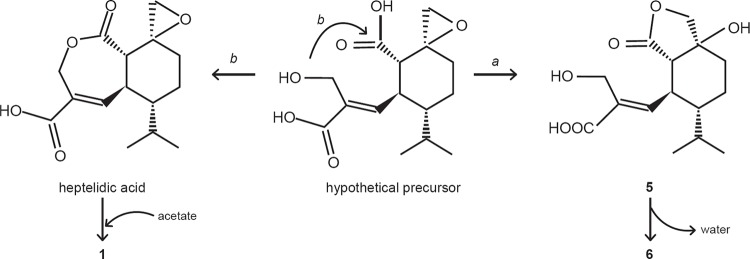
hypothetical biosynthetic precursor to explain the formation of compounds 1, 5, 6, and heptelidic acid.

Considering compounds 1, 5, 6 (Fig. 2), and heptelidic acid (Fig. 3), we hypothesise that they originate from a putative common precursor that could cyclise in two different ways to form five and seven membered lactone rings (Fig. 3, routes *a* and *b*, respectively). Compound 1 could be formed by opening of the epoxy ring of heptelidic acid by an acetoxy group (Fig. 3), while 2 and 3 are close variants of piliformic acid. According to Arigoni (1975), who investigated the biosynthesis of heptelidic acid, this compound was originally isolated from the ascomycete *Anthostoma avocetta* at Sandoz Pharmaceuticals (Sandoz AG) in 1971 and was named avocettin. The first total synthesis of heptelidic acid was achieved by the Danishefsky group in 1988 (Danishefsky & Mantlo 1988). Recently, Rahier et al. (2015) reported the large-scale production of heptelidic acid and the semi synthesis of several derivatives for structure activity relationship (SAR) studies using more than 100 cancer cell lines and *in vivo* and pharmacokinetics analysis in tumour xenographs. They concluded that heptelidic acid is a non-selective cytotoxic agent (IC_50_ values between 0.4 and 3 µM) that displays very modest activity *in vivo* and, as a consequence, is not a viable option for human cancer therapy. They also concluded, based on the SAR results, that both the epoxide and the seven-member lactone rings are essential for bioactivity. In our experiments, the novel natural product (1), in which the epoxide ring of heptelidic acid was opened, was active in the assays with *L. (V.) braziliensis*. Hydroheptelidic acid (5), in which the epoxide was absent and the 7-membered lactone ring was substituted by a 5-membered lactone, showed activity against amastigote forms of *L. (V.) braziliensis*. Interestingly, if compound 5 eliminated a water molecule to form compound 6, the activity against *L. (V.) braziliensis* was lost (Table V).

Piliformic acid (4) was previously isolated from *Hypoxylon deustum* (Anderson et al. 1985) and reported to be toxic to KB cells (derived from human mouth epidermoid carcinoma) and BC-1 human breast cancer cells with IC_50_ values of 13 µg/mL and 5 µg/mL, respectively. In addition, piliformic acid isolated from *Xylaria* sp. strain CY-6884 was considered inactive at 25 nM when tested against *Plasmodium falciparum* (3D7) and A549 cells (adenocarcinomic human alveolar epithelial cells) (Calcul et al. 2013).

In the current investigation, two new derivatives of piliformic acid with different moieties at the end of the alkyl side chain, 6′-acetoxypiliformic acid and 5′,6′-dehydropiliformic acid, were isolated. Among them, 6′-acetoxypiliformic acid (2) demonstrated better antileishmanial activity when compared to piliformic acid (4), while 5′,6′-dehydropiliformic acid was not active (Table V). Based on the results with *L. (V.) braziliensis* LUC, we suggest that the moiety at the end of the alkyl side chain influences the antileishmanial activity.

Although cytochalasin D (7) showed high IC_5O_ value against *L. (V.) braziliensis* in our assays, Roy et al. (2014) showed that this compound is able to destabilise the actin cytoskeleton of macrophages and reduce the attachment of *Leishmania* promastigotes and the intracellular amastigote load. These results reveal that the host actin cytoskeleton is responsible for parasite entry and infection. Another compound from the same class, 18-des-hydroxy cytochalasin H, isolated from the ethyl acetate extract from the endophytic fungus *Diaporthe phaseolorum*-92C, reduces the viability of *L. amazonensis* promastigotes (IC_50_ of 9.2 µg/mL) (Brissow et al. 2017). Cytochalasins induce inhibition of cellular motility by affecting the rate of actin polymerisation; this is a promising strategy for the discovery of new drugs to treat leishmaniasis (Roy et al. 2014).

We evaluated the cytotoxic activity of isolated compounds (1-7) on Vero cells, a normal line of kidney fibroblasts from African green monkey. The cytotoxicity was also assessed on THP-1, a human leukaemia monocytic cell line that has been extensively used to study monocyte/macrophage functions (Chanput et al. 2014). Vero cells are generally more sensitive (Bueno et al. 2011) and are often used to assess toxicity in antitumor tests because they are not derived from tumours. Although the lack of aqueous solubility limited the use of higher concentrations of the isolated compounds in the assays with mammalian cells, our results demonstrate that all compounds were more selective to the parasite.

The Lipinski rules were established for oral administration of drugs based on the physicochemical profiles of phase II drugs indicating their solubility and permeability (Lipinski et al. 2001). The isolated compounds (1-7) satisfied some Lipinski simplified rules such as having a molecular weight (MW) ≤ 500 or the logarithm of the octanol-water partition coefficient, log *P*(o/w) is < 5. The log *P* values of the isolated compounds were between -1 and 3 (Supplementary data, Table), similar to several drugs in clinical use (pentamidine, miltefosine, and amphotericin B) to treat leishmaniasis.

Most of the active compounds (1, 2, 4, and 5) have acid moieties in their structures with the possibility of interand intramolecular interactions through hydrogen bonds, which make these compounds slightly ionisable. However, less-ionisable compounds tend to permeate more easily in the membrane. Although natural products can violate some rules, the confirmatory test against intracellular amastigotes showed their potential as anti-parasitic drugs.

The Molinspiration Cheminformatics software was used to predict bioactivity of isolated compounds. Compounds 1, 5, and 6 had the best scores, suggesting their ability to act as enzyme inhibitors (Supplementary data, Table). The molecular docking analysis to test the isolated bioactive compounds toward target proteins from *Leishmania* spp. could help us to elucidate their mechanisms of action. However, there are more than 52 different protein structures of *Leishmania* spp. and new investigations are necessary to guide future experiments (Ogungbe et al. 2014).

In a search carried out in the SciFinder Scholar (searched on July 26, 2017), few studies have reported compounds isolated from fungal sources with antileishmanial activity. We highlight the compounds terrain, butyrolactone I, and butyrolactone V, isolated from *Aspergillus terreus*-F7, an endophytic fungus from *Hyptis suaveolens* (L.) Poit, which showed antileishmanial activity against *L. amazonensis* with IC_50_ values from 23.7 to 78.6 µM and low cytotoxicity toward the normal cell line GM07492A (IC_50_ values from 3.4 × 10^3^ to 15.3 × 10^3^ µM) (da Silva et al. 2017). Thus, the search for bioactive natural products is an alternative for seeking new scaffolds aimed at developing antileishmanial drugs.

In summary, this is the first report on the chemical investigation of *N. pseudotrichia* as a source of novel natural products with activity against intracellular amastigote forms of *L. (V.) braziliensis* and relative non-toxicity to mammalian cells.
