# Supplementary material for: Leishmanicidal compounds of Nectria pseudotrichia, an endophytic fungus isolated from the plant Caesalpinia echinata (Brazilwood)
Source: Mem Inst Oswaldo Cruz. 2018 Feb;113(2):102–10. doi: 10.1590/0074-02760170217 (PMC5722265; doi:10.1590/0074-02760170217)
Supplement: Supplementary file 1 [file 0074-0276-mioc-113-02-0102-Suppl01.pdf]

Mem Inst Oswaldo Cruz, Rio de Janeiro, Vol. *113*(2): 1-1, February 2018

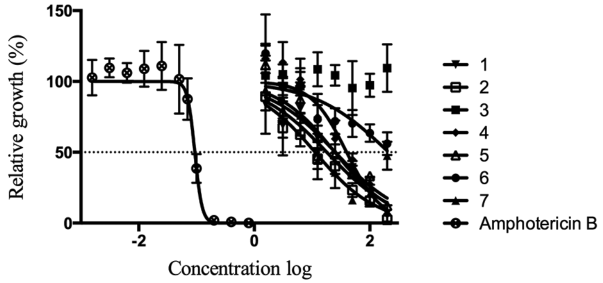

Fig. 1: concentration-response curves of isolated compounds (1-7) and amphotericin B on *Leishmania (Viannia) braziliensis* expressing firefly luciferase as a reporter gene. Compounds 1, 2, and 5 were more active, with IC_{50} values of 21.4, 28.3, and 24.8 µM, respectively. Compounds 4 and 7 showed IC_{50} values of 78.5 and 72.6 µM, respectively, and amphotericin B showed IC_{50} of 0.12 µM. Luciferase activity was measured by luminescence detection after 72 h treatment. IC_{50} values were calculated using GraphPad Prism (Version 6.0c).

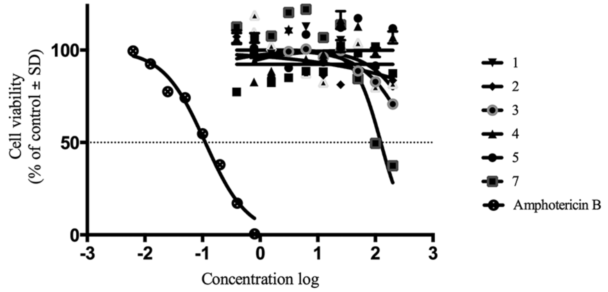

Fig. 2: concentration-response curves of isolated compounds (1-7) and amphotericin B on THP-1 cells. None of these compounds were considered toxic to THP-1 cells. Amphotericin B showed IC_{50} of 12.0 µM in the assay on THP-1 cells. Cell viability was quantified by MTT after 72 h treatment and IC_{50} was calculated using GraphPad Prism (Version 6.0c).

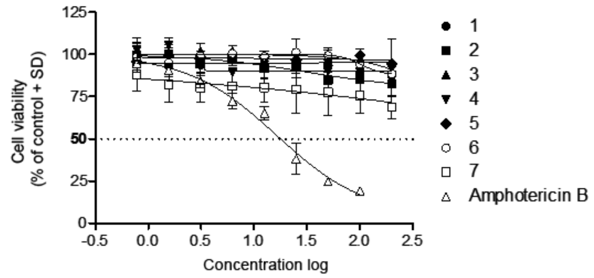

Fig. 3: concentration-response curves of isolated compounds (1-7) and amphotericin B on Vero cells. None of these compounds were considered toxic to Vero cells. Amphotericin B showed IC_{50} of 18.2 µM in the assay on Vero cells. Cell viability was quantified by MTT and IC_{50} was calculated using GraphPad Prism® Version 5.01.

TABLE

Table of log *P* values and predicted bioactivity scores

Log P					Bioactivity calculated score [C]					
Compound	[A]	[B]	[C]	[D-F]	GPCR ligand	Ion channel modulator	Kinase inhibitor	Nuclear receptor ligand	Protease inhibitor	Enzime inhibitor
1	2.63	1.35	1.24	-	low	0.24	low	0.45	low	0.41
2	2.37	2.80	2.28	-	low	low	low	0.29	low	0.34
3	2.17	3.00	1.95	-	low	low	low	low	low	0.20
4	2.69	3.38	2.63	2.83^{D}	low	low	low	low	low	0.24
5	0.78	0.58	0.74	-0.83^{D}	low	low	low	0.52	low	0.53
6	1.13	1.65	1.13	1.63^{D}	low	0.34	low	low	low	0.54
7	2.89	2.64	2.89	3.04^{D}	low	low	low	low	low	0.34
Pentamidine	-	-	-	4^{E}; 2.32^{F}; 1.32^{F}	-	-	-	-	-	-
Miltefosine	-	-	-	2.68^{F}; 2.25^{F}	-	-	-	-	-	-
Amphotericin B	-	-	-	0.8^{E}; -0.66^{F}; -2.3^{F}	-	-	-	-	-	

A: Tetko et al. (2005); B: Advanced Chemistry Development Inc., 2016. (ACD/Labs) ACD/PERCEPTA Version 2015. Frankfurt am Main. Available from: www.acdlabs.com; C: Molinspiration Cheminformatics 2017. Available from: http://www.molinspiration.com/cgi-bin/properties; D: SciFinder Database. Available from: https://scifinder.cas.org; E: DrugBank Database, DrugBank Version 5.0. Available from: https://www.drugbank.ca/. Experimental property; F: drugBank Database, DrugBank Version 5.0. Available from: https://www.drugbank.ca/. Predicted property.
